# The relationship between autistic traits, expressiveness, readability and social perceptions

**DOI:** 10.1371/journal.pone.0301003

**Published:** 2024-03-28

**Authors:** Rabi Samil Alkhaldi, Elizabeth Sheppard, Zack Ellerby, Emily Rachel Reed Burdett, Peter Mitchell

**Affiliations:** 1 School of Psychology, University of Nottingham, Nottingham, United Kingdom; 2 Taif University, Taif, Saudi Arabia; 3 School of Computer Science, University of Nottingham, Nottingham, United Kingdom; 4 School of Social Sciences, University of Bradford, Bradford, United Kingdom; Southeast University - Jiulonghu Campus, CHINA

## Abstract

This study investigated the relationship between autistic traits, expressiveness, readability (both actual and perceived), social favourability, and likability. Sixty participants designated as ‘targets’ were video recorded in a range of social scenarios and their autistic traits were measured using the Autism Spectrum Quotient. The videos were then shown to 106 new participants designated ‘perceivers’, who were split into three groups to make judgments related to readability, expressiveness, and social favourability respectively. Mediation analyses revealed that autistic traits negatively impacted both perceived likeability and social favourability, mediated by lowered expressiveness. Autistic traits also directly impacted readability, which was not mediated by expressiveness. The findings show how the level of autistic traits of a target can influence how they are socially perceived by others.

## Introduction

The way in which an individual is perceived by unfamiliar others can have far-reaching implications. For instance, first impressions have been found to affect employment opportunities [1], voting in elections [2], and criminal justice decisions [3]. People are more likely to collaborate with individuals who are perceived as more socially warm [4], and more likely to judge it as acceptable to socially exclude those perceived as socially cold [5]. Past research has extensively investigated the impact of aspects of a person’s (a “target’s”) physical appearance on these impressions [6, 7], demonstrating that targets with more physically attractive faces tend to be perceived more favourably across a range of personality characteristics as well as being more liked [8, 9].

Another factor that may influence initial impressions of interpersonal liking is how easy a target’s behaviour (facial expressions and body movements) is to read. Anders et al. [10] established that perceivers found other individuals (targets) more likeable, when they felt that they (the perceivers) were more accurate at reading their expressions. However, as this study focused only on perceived readability i.e. whether the perceiver thought they had read the target’s expression rather than objectively measured readability, e.g., whether they had correctly identified the expression, the possibility remains that the effect was driven by a ‘halo’-type effect [11], whereby targets perceived as more likeable were simply perceived as being more readable, irrespective of whether this is actually true. The study was further limited by only including female targets and readability impressions were based on only two types of facial expression.

An association between readability and social favourability has also been observed in targets who are autistic [12]. Autism is a developmental disability which is diagnosed based on difficulties in social communication and restricted interests/repetitive behaviours, and may or may not occur alongside other developmental disabilities or intellectual disability (see [13] for full diagnostic criteria). While historically the difficulties in social communication have been attributed to a deficit in mindreading in autistic people [14], recent research has established that autistic people’s behaviour is less readable by their non-autistic peers [15], and they are also judged less socially favourable than non-autistic people based on brief samples of their behaviour, [16, 17]. Collectively these findings implicate the important role of the perceptions of non-autistic interaction partners in the social disability experienced by autistic people [18]. Understanding these mechanisms is particularly important given that autistic people experience a range of negative social outcomes such as higher rates of mental health difficulties [19] and increased loneliness [20] in comparison to non-autisic people, and challenges around employment [21].

Based on these observations as well as the findings of Anders et al. [10], Alkhaldi et al. [12] questioned whether there is an association between being less readable and being viewed as less socially favourable amongst autistic targets (as well as non-autistic targets). They utilised natural videos taken from an earlier study [15] in which targets reacted to one of four scripted event scenarios enacted by the researcher, such as telling a joke or paying the target some compliments. Actual target readability was calculated based on the number of perceivers who correctly guessed which event the target reacted to. A correlation was found between readability and social favourability i.e. those targets who perceivers found more difficult to read, also tended to be those who were perceived less favourably. The autistic targets were both less readable by perceivers (who were non-autistic) and rated as less socially favourable. The correlation between readability and social favourability held even when controlling statistically for diagnostic status, implying a transdiagnostic effect.

While the findings of Alkhaldi et al. [12] are consistent with the possibility of an association between actual readability and social favourability, there were a number of limitations of the study. Firstly, each target only experienced one of four scenarios, and this was used as the basis for readability measurements. This meant that measurements of target readability and social favourability were contaminated by scenario, making it challenging to compare scores for targets who did not happen to experience the same scenario. Secondly, it was not clear why the relationship emerged between readability and social favourability or indeed whether there is a direct causal relationship between these two variables. Anders et al. [10] put forward a “reward” explanation for their findings, arguing that when a perceiver feels that they have accurately read the target, this creates a positive association towards that particular target, resulting in a feeling of liking. However, in relation to Alkhaldi et al. [12] other explanations are possible, as acknowledged by the authors. For instance, it could be that perceivers are less motivated to read targets who are less socially favourable such that lower readability is actually a consequence of lower social favourability as opposed to the reverse. Alternatively, it could be that some third variable impacts both readability and social favourability leading to an apparent association between the two.

One variable that could plausibly explain the relationship between readability and perceived social favourability of a target is how expressive the target is in their behaviour. If a target is less expressive in their behaviour, then we can assume that they produce a weaker “signal” to be read, making it likely that perceivers would find it more difficult to interpret their behaviour [22]. A less expressive target might also be perceived as less ‘open’, more secretive, or more introverted [23]–and introverts also tend to be perceived less socially favourably [24]. Hence expressiveness might independently predict both readability and social favourability of targets, giving rise to an indirect association without there being any direct relationship between the two.

Another variable that might account for the relationship between readability and perceived social favourability is the level of autistic traits that an individual has. It has been proposed that autistic traits exist on a continuum within the population, at varying levels within both diagnosed autistic and non-autistic people [25, 26]. Unlike an autism diagnosis, which requires observation and clinical judgment, autistic traits are typically measured using self-report questionnaires such as the Autism Spectrum Quotient (AQ) [25]. The AQ asks about a person’s engagement in a wide range of behaviours characteristic of those diagnosed as autistic, including differences in social interaction and communication. Higher scores on the AQ might reflect the person engaging in less typical or normative social behaviour, which may in turn be more difficult for others to read [15], and might be perceived as less socially favourable [27]. However, so far no previous studies have directly investigated whether autistic traits impact how individuals are perceived by others.

Autistic traits and expressiveness may also be related. Some evidence suggests that diagnosed autistic people are less expressive than non-autistic people, although findings are mixed. It has been reported that autistic people produce facial expressions less frequently and for shorter duration than non-autistic people [28–30]. A meta-analysis [31] revealed that autistic people are less likely to share facial expressions with others and tend not to imitate the expressions of faces or face-like stimuli. Elsewhere, however, autistic people are reported to be as expressive as non-autistic people during automatic imitation [32–34], and while retelling stories [35]. In addition, when the expression is based on real social interaction, autistic people are rated just as expressive as non-autistic people in different scenarios [15, 36–38], leading to the suggestion that autistic people may be equally expressive but in non-typical ways [15]. However, to our knowledge, no research has yet examined whether level of autistic traits relates to expressiveness. Therefore, the role of expressiveness in relation to readability, social favourability and autistic traits requires further investigation.

A key aim of the research outlined in this paper was to test the hypothesis that there is a positive association between target readability and social favourability, overcoming some of the limitations of previous research in this area. Readability and social favourability of targets was measured over a range of scenarios, enabling a more comprehensive measure of each construct and allowing direct comparability between targets. As in previous research, social favourability was measured by asking perceivers to rate targets on a range of socially desirable characteristics [12, 16], while in addition a more direct measure of perceivers’ attitudes to the target was taken by asking perceivers to judge whether or not they liked the target [39]. By including both social favourability and the likeability as outcome measures we were able to determine whether scores on social favourability trait measures are tantamount to being a measure of liking.

As one of the two previous studies focused on actual readability i.e. how well perceivers could interpret the targets’ behaviour [15] and one on subjective impressions of readability i.e. whether perceivers believed that they could interpret the targets’ behaviour [10], in the current research both were measured, allowing us to determine the relationship between them and contribution of each to social favourability/likeability scores. In line with previous research [10], we expect that any association between actual readability and social favourability will be explained by perceived (subjective impressions of) readability. The perceived expressiveness of each target was also measured to explore the possibility that expressiveness might impact both readability and social favourability, or even account for any observed relationship between the two. Finally, we examined the impact of levels of autistic traits on all variables in the model.

**Fig 1:**
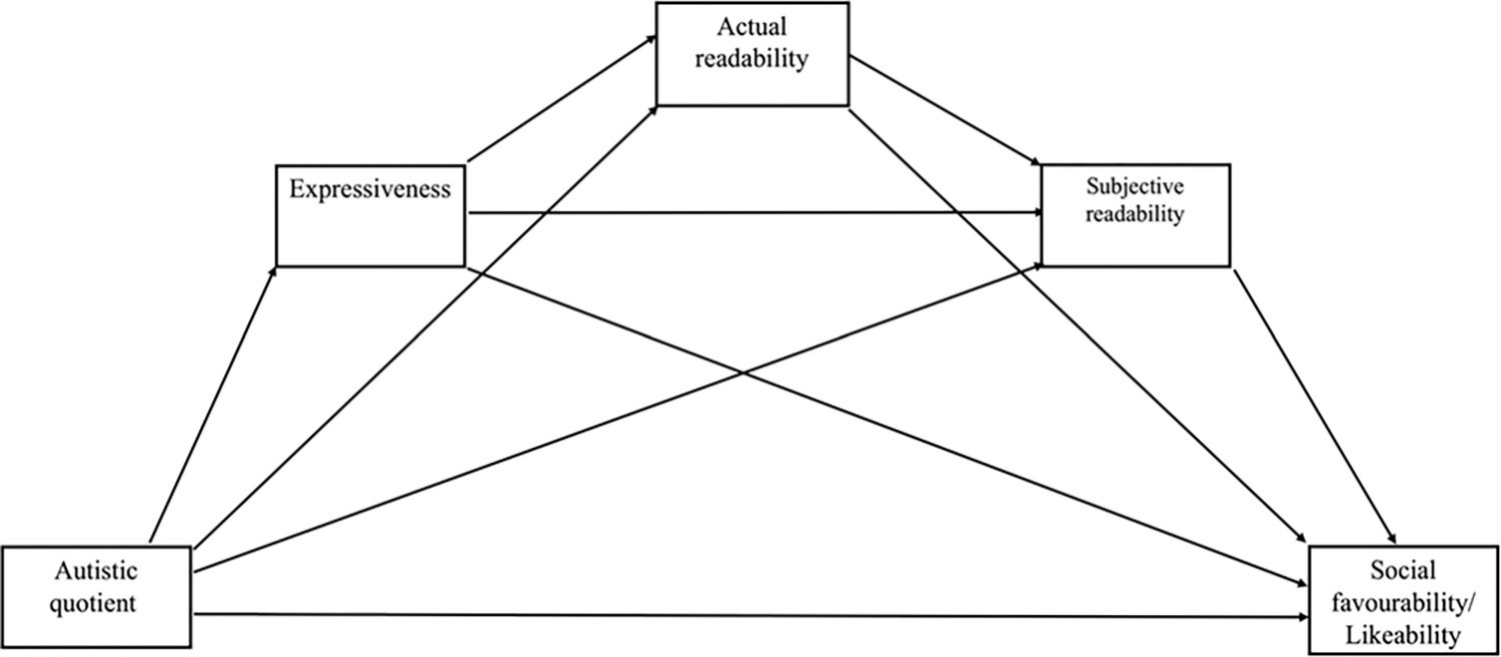
Mediation model positing predictor variables and outcome variables.

The study used mediation analysis to examine the a priori model displayed in Fig 1, which hypothesises that those high in autistic traits (i.e., with higher AQ scores) may be perceived less socially favourably because of three putative mediating factors—expressiveness, actual readability, and perceived readability. Specifically, autistic traits are included as the predictor in the model as we assume them to be a characteristic of the target that contributes to their observable behaviour [25]. Based on the above review we predict that those with higher autistic traits will be less expressive in their behaviour [28]. Reduced expressiveness is expected to lead to lower actual readability as the target’s behavioural signal will be reduced. It is expected that lower actual readability will be associated with lower perceived readability insofar as perceivers are aware when the target is difficult to read [10]. Finally, we expect lower perceived readability and lower expressiveness to be associated with lower social favourability and likeability.

## Methods

The entire procedure was approved by the Ethics Committee, [Institution redacted for review purposes] (Ethics approval number: S1178R). Informed consent was obtained from all participants.

The full procedure consisted of a stimulus development phase in which video recordings and AQ scores of targets were obtained, followed by a perceiver phase in which three different groups of perceivers viewed the videos to capture the following measures for each target: 1) readability (actual target readability i.e. how accurately perceivers identified which scenario the target was reacting to; and subjective target readability i.e. how readable the perceivers judged the target to be) 2) target expressiveness as judged by the perceivers; and 3) target social favourability/likeability as judged by the perceivers.

### Stimulus development

In this stage we created video stimuli to be used in the main experiment. The participants (hitherto referred to as “targets”) were secretly video recorded responding to four different aspects of the researcher’s behaviour. The scenarios (named the Story scenario, Negative Feedback scenario, Gift scenario and Debriefing scenario) were designed in line with principles and/or used in previous research with this task to assess readability [40, 41]. In short, we aimed to create events that would provoke a reaction but would be unlikely to cause a major disturbance in the mood of the participant. Scenarios also needed to be plausible within the context of an experiment, and to be suitable for the experimenter to enact convincingly. None of the scenarios were intended to elicit a specific emotion, as explained in [42]. Further details appear below.

#### Participants (targets)

A total of 60 targets were recruited (33 males and 27 females). Six males and three females disclosed that they had been formally diagnosed by a clinician as being autistic. Participants were recruited using the ‘participant recruitment system’ and advertisements at [Institution redacted for review purposes]. The majority of targets were White British (54), and there were 3 Black British, 2 Asian (1 Chinese and 1 Malaysian) and 1 Brazilian. All participants were university students but specific information on socio-economic status was not recorded. The researcher administered Wechsler Abbreviated Scales of Intelligence (WASI, [43]), primarily as a decoy task, but also to determine whether any diagnosed autistic participants had intellectual disability, and details appear in Table 1.

**Table 1:** Target characteristics.

	*M*	*SD*	*Range*
Age (Years)	19.48	1.68	18–26
Full-scale IQ	104.55	4.70	92–115
AQ	22.52	9.22	7–46

#### Materials and apparatus

A room within the School of Psychology was used. A Panasonic HC-W580 Camcorder (2.51 MP, FHD, Wi-Fi, 3-inch LCD) on a tripod was placed approximately 1.2m directly opposite the target’s seating position, across the table. The camera was positioned so that the participant’s face, neck, shoulders, and chest could be seen.

All targets completed the 50-item Autism Spectrum Quotient (AQ) [25] as a measure of autistic traits. The AQ is a self-report questionnaire in which the participant is asked to rate themselves on a wide variety of characteristics associated with autism. Participants receive a score out of 50 depending on how many items they agree apply to them, with reverse scoring where needed. The AQ cannot be used for autism diagnosis, but can be used as a screener, with a score of over 32 considered to warrant further investigation.

All participants completed the WASI [43] which consists of four subtests, two (vocabulary and similarities) measuring verbal ability, and two (block design and matrix reasoning) measuring non-verbal ability. All subtests were administered and scored in accordance with the manual instructions yielding a composite measure of full-scale IQ.

#### Procedure

Each target arrived individually at the testing room and was asked if it would be okay to switch on the camera later to film them when it was time to do the main task of problem solving. In reality though, the camera was turned on before targets entered the room. Targets were asked to read the information sheet, which included, “The aim of this experiment is to investigate how people perform a taxing activity. You will be given a problem-solving task. When the time comes, I will switch on this camera to record how you tackle the problems”. After the target sat down, the researcher provided the context (*the story scenario*);

“*Before we start*, *let me tell you a little bit about myself*: *I’m Rabi and I obtained my undergraduate degree from a school of education in Saudi Arabia*, *and then I was sent to the UK to study for my Masters and PhD*. *It is a great opportunity for me but the big problem I face is that it is very difficult to communicate with people because I use English as a second language and I’m from a different culture*. *Sometimes I feel quite isolated and lonely–it’s difficult to make friends here”*.

Subsequently the researcher said *“OK*, *let’s start the task and I will turn the camera on*.*”* Then the target tackled the WASI, ending with Block Design, which served as the ‘problem-solving task’. When he or she finished, the researcher pretended to switch off the camera and said (negative feedback scenario): *“I can see you found this task (Block Design) really difficult*. *You took quite some time to complete the puzzles; most of the other participants performed this task much more quickly*.*”* The researcher paused for a few seconds, looking down and said (gift scenario): *“Anyway*, *I very much appreciate your time and participation*. *As an extra ‘thank you’ I’d like to offer you a gift*. *I have something in this bag I wish to offer you*.*”* After offering the gift, the researcher paused for a few seconds looking at the target and said (debriefing scenario): *“You have been recorded by the camera for the whole time since you entered this room and the camera is still recording; now*, *I am going to switch off the camera*.*”*

Targets were then fully debriefed about the true purpose of the study (i.e. to capture their natural expressions while interacting with the researcher and what their videos would be used for. All targets consented to the use of their videos. This phase of the study took approximately 40 minutes for each target, and participants were compensated with an inconvenience allowance or given course credit.

#### Video editing

The face and upper body of each target was visible in the videos. Video recordings were edited with I-Movie software following methods developed in previous research [15, 40, 44]. In order to do this, the researcher used a set of criteria to edit the video recordings to ensure objective selection of video excerpts across the target groups. The aim was to generate video clips for each scenario capturing the timeframe when the targets were likely to be most expressive. The recordings for the *story scenario* were edited from the point where the researcher said, “…. because I use English as …” to “… it’s difficult to make friends here”. *The negative feedback scenario* was captured from the point when the researcher gave the target negative feedback on the block design task saying “I can see you found this task (block design) really difficult…” to “…most of the other participants performed much more quickly”. *The gift scenario* was edited from the point when the researcher raised the gift bag from the floor and presented it to the target, saying, “I’d like to offer you a gift…” to “…I wish to offer you…” *The debriefing scenario* was edited from the point where the researcher said, “you have been recorded…” to “…and the camera is still recording.” This resulted in a total of 240 edited video clips. The dimensions of the video clips were 1920 pixels in width and 1080 pixels in height, presented at 25 frames per second. The mean lengths of the edited videos in seconds were: 5.90 (story scenario), 5.82 (negative feedback scenario), 4.05 (gift scenario) and 4.55 (debriefing scenario). As previous studies [15, 40, 44] target vocalizations (i.e. the auditory component of footage) were muted.

### Perceiver phase

For each target there were four videos displaying their reactions to each scenario (listening to the experimenter’s story, receiving negative feedback, receiving a gift, and the debrief). The four videos generated by each target were presented simultaneously, playing on a loop, one in each quadrant of the screen. This format was used (as opposed to presenting each video individually) so that perceivers’ judgments could be informed by multiple samples of target behaviour simultaneously, to provide a global judgment about the target.

The videos were shown to different sets of non-autistic perceivers across three independent waves of data collection (focusing on readability, expressiveness and social favourability respectively) as detailed in the following sections.

#### Wave 1: Readability

The aim of this wave of data collection was to measure readability (both actual and subjective readability).

#### Participants

Forty-six non-autistic perceivers (31 females and 15 males) aged between 18 and 27 (*M* = 20.96 years, *SD* = 3.07), were recruited through the ‘participant recruitment system’ and through advertisements at [institution redacted for review purposes]. Adverts specified that perceivers should be “neurotypical” and all perceivers confirmed verbally that they were not autistic. Specific data on race and socioeconomic status was not recorded. The number of perceivers was based on previous similar studies [12, 15]; notably, all measures represent averages across perceivers as the target was the unit of analysis.

#### Procedure

All videos from all 60 targets were shown to each perceiver on a 21.5-inch iMac, with each target presented in random order using PsychoPy3 version 3.2.5 [45]. Each perceiver (tested individually) viewed video clips of targets experiencing the four scenarios (listening to the experimenter’s story, receiving negative feedback, receiving a gift and the debrief scenario). On each screen, the perceiver viewed four videos of the same target (as they reacted to each of the four scenarios), all played in a loop. Both the order of the targets and the location of the four scenarios on the screen was randomised. The four scenario names were listed beside each video and perceivers were instructed to decide which scenario the target was experiencing in each video. They were instructed not to use a given scenario name for more than one video. As all four videos appeared on the screen simultaneously, participants were able to label the videos in any order they chose, could directly compare videos, and could alter their decisions at any point while they remained on that particular screen. After applying labels to each of the four videos, perceivers were able to press the <next> button to go to the next screen which presented the same videos with a single question “How readable is this person?” (This was expressed on a scale of 1–7, where 1 = not readable and 7 = highly readable.) Participants answered this question by clicking the appropriate number from 1 to 7 based on their opinion. Perceivers were not informed that any of the targets were autistic.

#### Wave 2: Expressiveness

The aim of this wave of data collection was to measure perceived levels of target expressiveness.

#### Participants

Thirty newly recruited non-autistic perceivers (19 females and 11 males) aged between 18 and 24 (*M* = 19.42 years, *SD* = 1.94), were recruited through the ‘participant recruitment system’ and advertisements at [institution redacted for review purposes]. As previously, all perceivers confirmed verbally that they were not autistic.

#### Procedure

All four videos appeared on the screen simultaneously and were repeated on a loop, as described above; perceivers were not told anything about the scenarios that targets were reacting to and neither were they told that some targets were autistic. Perceivers were asked to rate targets on how ‘expressive’ they were on a 7-point scale from 1–7 (where 1 is ‘not expressive’ and 7 is ‘highly expressive’). As perceivers made a single judgment for each target, they could take into account information from all four videos. Perceivers responded to the question appearing in the middle of the screen while the target videos remained visible.

#### Wave 3: Social favourability

This wave of data collection measured perceived social favourability and likeability.

#### Participants

Thirty newly recruited, non-autistic perceivers (18 females and 12 males) aged between 18 and 27 (*M* = 20.96 years, *SD* = 3.07), were recruited through the ‘participant recruitment system’ and advertisements at [institution redacted for review purposes]. As previously, all perceivers confirmed verbally that they were not autistic.

#### Procedure

The procedure, stimulus presentation and response mode were all identical to the readability and expressivity waves of data collection, but in this case perceivers rated each target on a 6-point scale from 1–6 (where 1 was low and 6 was high) in response to on-screen questions presented in a fixed order: How awkward is this person? How trustworthy is this person? How empathic is this person? How would you rate this person’s self-esteem? A final question requested an overall opinion: Do you like this person; Yes or No? All questions appeared on-screen one after another and the target videos remained on screen while the perceivers answered the questions listed above. Perceivers were prompted to press the ‘next’ button to proceed to the next question until they had completed all questions for each target.

The social favourability scale used questions from a 9-item scale that has been used extensively in previous research [12, 16, 17]. To reduce burden on participants only five items were used as previous research has revealed extremely high correlations between such questions. The five questions were selected based on pilot work in which perceivers judged which of the nine social favourability items were most important in deciding whether they liked or disliked each target [39]. Perceivers were not informed that any of the targets were autistic.

## Results

### Data handling

A number of dependent variables were extracted from the data as defined in Table 2, which also reports the mean scores (and standard deviations) for each variable. The unit of analysis was the target, so all variables represent means (across all perceivers). See **S1 Table** for details of accuracy for each scenario.

**Table 2:** Definitions of each variable, with mean scores and standard deviations.

Dependent Variable	Item Definition	*M*	*SD*
Actual target readability	Mean proportion of scenario reactions correctly identified (across all perceivers) for each target	0.54	0.10
Subjective target readability	Mean subjective readability as a rating out of 7 across all perceivers based on their judgment of “How readable is the target?”	4.09	0.83
Target expressiveness	Mean expressivity rating (across all perceivers) associated with each target, as rated out of 7.	3.73	1.01
Target social favourability	Mean of the sum of the rating of four items: awkwardness (reverse-scored), trustworthiness, empathy and self-esteem (across all perceivers)	3.93	0.56
Target likeability	Number of “likes” that each target received (across all perceivers) based on a binary rating	23.1 (4.78)	

### Preliminary analyses

The key aim of this study was to investigate whether autistic traits have a negative association with social favourability and/or likeability, and if so, whether this is associated with target readability and expressiveness. Before proceeding to examine the specific pattern of relationships between key variables hypothesised to impact social favourability, initial analyses were conducted to assess the correlations among all variables. Here, we report results of both zero-order and partial correlations, controlling for the effect of autism diagnostic status of targets as a covariate (9 targets were diagnosed with autism). All correlation coefficients (Pearson’s *r*) are reported in Table 3.

**Table 3:** Pearson inter-correlations between all key variables: Zero order and correlations with autism diagnosis partialled out.

Variable	2.	3.	4.	5.	6.
**1. Actual target readability**	.51***	.30*	.33*	.28*	-.43**
**Partial**	50***	.32*	.32*	.31*	-.35**
**2. Subjective readability**	-	.75***	.78***	.86**	*-*.46***
**Partial**	-	.75***	.78***	.87**	*-*.50***
**3. Target social favourability**	-	-	.82***	.80***	*-*.38**
**Partial**	-	-	.82***	.80***	-.47***
**4. Target likeability**	-	-	-	.80***	-.29*
**Partial**	-	-	-	.80***	-.30*
**5.Target expressiveness**	-	-	-	-	-.34**
**Partial**	-	-	-	-	-.45***
**6. Target AQ**	-	-	-	-	-
**Partial**	-	-	-	-	-

*p < 0.05

** *p* < 0.01

***p < 0.001

Including all targets in the analysis, actual target readability correlated positively with subjective readability. In addition, actual readability significantly correlated positively with social favourability and likeability, although to a much lesser extent than did subjective readability. Further, categorical liking was strongly associated with social favourability. Rated expressivity also correlated significantly and positively with all other variables. AQ scores, on the other hand correlated significantly and negatively with all variables, such that higher AQ scorers had lower actual readability, were rated as less expressive, less readable and perceived as less socially favourable and likeable. To determine whether the observed relationships held irrespective of a diagnosis of autism, the same analyses were conducted while controlling for autism diagnostic status of participants as a covariate. Findings were substantially the same as zero-order correlations, with significant positive relationships observed between expressiveness, actual target readability, subjective readability, social favourability, and likeability, while AQ scores correlated negatively with all these variables.

### Mediation analyses

Mediation analysis was conducted using PROCESS macro for SPSS [46], using model number 6. This model was selected to assess the effect of AQ on social favourability and likeability (in separate models) with three putative mediating factors, organised according to a hypothesised directional pathway of effect through these mediators—i.e., autistic traits may impact expressiveness, which may impact actual readability, which may in turn impact perceived readability, and then social favourability and/or likeability. Effects were calculated for each of 10,000 bootstrapped samples. The aim of this analysis was to examine the pattern of relationships evident in the framework of our hypothetical model (shown in Fig 1). Specifically, we hypothesised that autistic traits (as measured with AQ), may influence perceived expressiveness, actual and subjective readability, and social favourability, as judged by perceivers—both directly or indirectly, via other mediating factors. Regarding order of mediators in the model, we reasoned that actual readability may be influenced by expressiveness but not vice versa, and that subjective readability may be affected by actual readability, insofar as the perceivers are able to sense that they could read the target’s mind, but not vice versa.

All values for each variable were converted to proportions ranging from 0–1: readability (hits divided by number of responses), subjective readability (standardised by subtracting 1 from the value and then dividing by 6 i.e., (x-1)/6), expressiveness (standardised by subtracting 1 from the value and then dividing by 6 i.e., (x-1)/6), social favourability (standardised by subtracting 4 from the value and then dividing by 20), likeability (standardised by dividing by 30, which was the number of perceivers), and AQ scores divided by 50.

**Fig 2:**
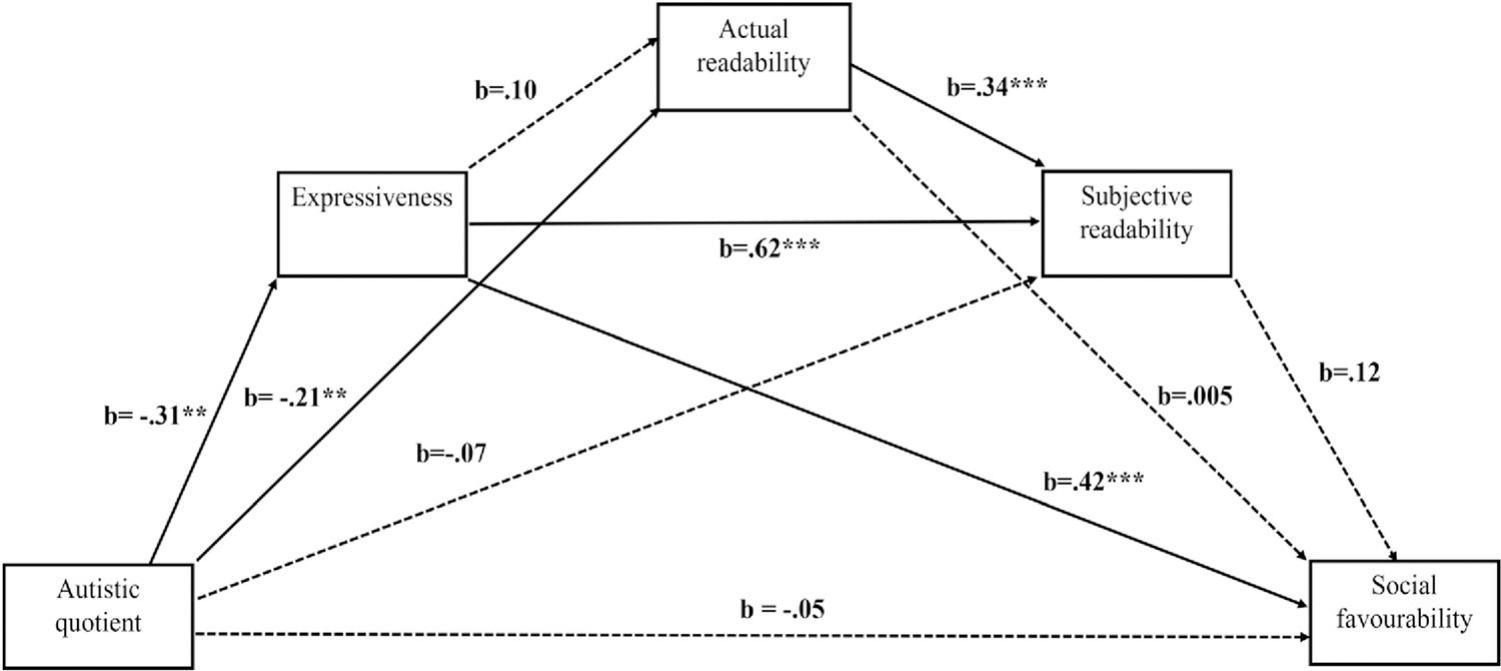
Mediation model for effects of AQ score on social favourability. Solid and dotted lines indicate significant and non-significant effects respectively (α < .05) *p < .05, **p < .01, ***p < .001.

Fig 2 shows the pattern of effects from AQ to the outcome variable of social favourability, via the three putative mediator variables. The total effect of AQ on social favourability was significant, *b* = -.22, *p* = .003 [-.37, -.08]. The direct effect of AQ on expressiveness was found to be significant, *t* (58) = -2.73, *p* = .008, with this predictor accounting for 11% of the sample variance (R^2^ = .11). The direct effect of AQ on actual readability was found to be significant, *t*(57) = -3.03, *p* = .004, but the effect of expressiveness on actual readability was nonsignificant, *t*(57) = 1.24, *p =* .22, with these two predictors accounting for 21% of the sample variance (R^2^ = .21). The direct effect of AQ on subjective readability was found to be non-significant, *t*(56) = -1.53, *p* = .13, while the effects of expressiveness, *t*(56) = 12.50, *p* < .001, and actual readability on subjective readability were both significant, *t*(56) = 4.03, *p* < .001, with these three predictors accounting for 82% of the sample variance (R^2^ = .82). The direct effects of AQ *t*(55) = -.96, *p =* .34, actual readability, *t*(55) = .05, *p* = .96, and subjective readability *t*(55) = .82, *p =* .42, on social favourability were each found to be nonsignificant; however, the effect of expressiveness on social favourability was significant, *t*(55) = 3.90, *p* < .001. Together, these four predictors accounted for 66% of the sample variance in social favourability (R^2^ = .66).

The indirect effects of AQ scores on social favourability were as follows: Through expressiveness, (-.31) (.42) = -.130, 95% CIs [−.26, -.03] (significant); through actual readability, (-.21) (.005) = -.001, 95% CIs [-.04, .03] (nonsignificant); through subjective readability, (-.07) (.12) = -.008, 95% CIs [-.04, .009] (nonsignificant); through expressiveness and actual readability, (-.31) (.10) (.005) = -.000, 95% CIs [-.007, .008] (nonsignificant); through expressiveness and subjective readability, (-.31) (.62) (.12) = -.023, 95% CIs [-.09, .03] (nonsignificant); through actual and subjective readability, (-.21) (.34) (.12) = -.009, 95% CIs [-.03, .009] (nonsignificant). Through expressiveness and both readabilities, (-.31) (.10) (.34) (.12) = -.001, 95% CIs [-.006, .003] (nonsignificant).

**Fig 3:**
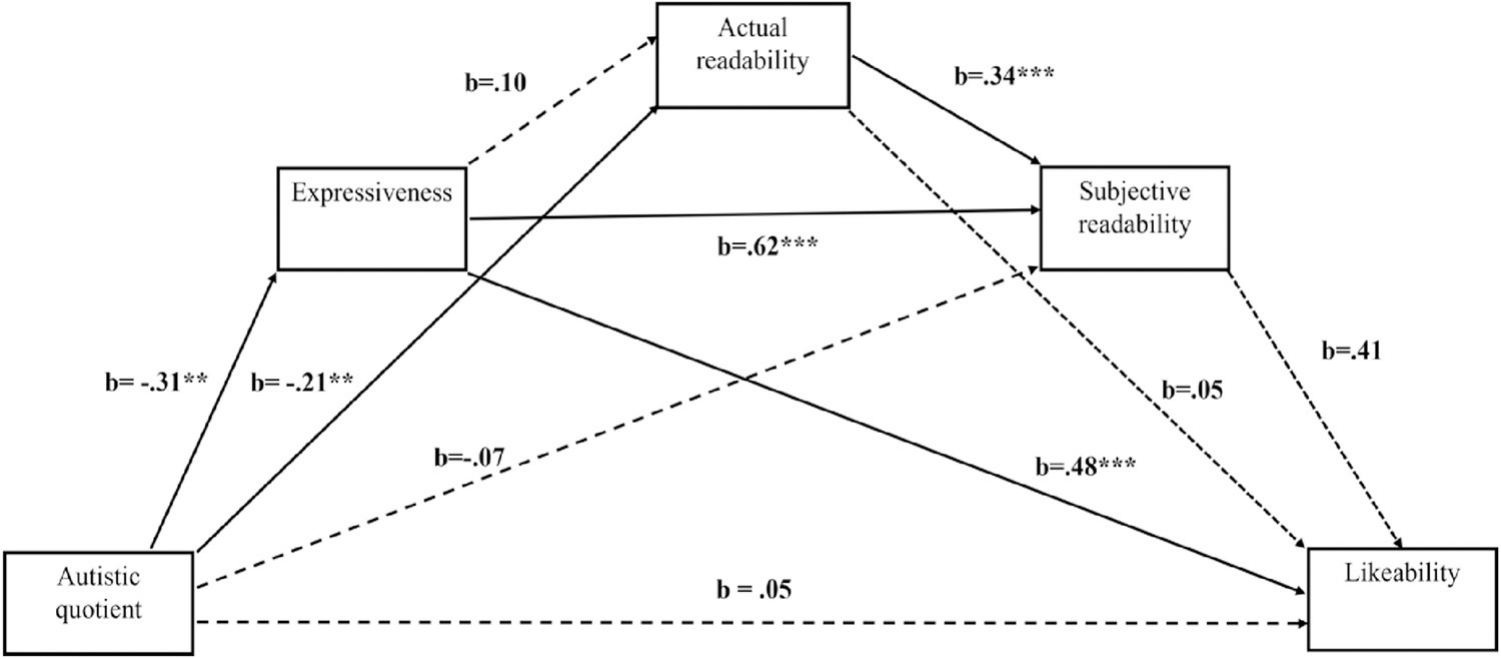
Mediation model for effects of AQ score on likeability. Solid and dotted lines indicate significant and non-significant effects respectively (α < .05) *p < .05, **p < .01, ***p < .001.

Fig 3 shows the pattern of effects from AQ to the outcome variable of likeability, including via the three putative mediator variables. The total effect of AQ on likeability was significant, *b* = -.25, *p* = .026 [-.47, -.03]. The direct effects of AQ *t*(55) = .65, *p* = .52, actual readability *t*(55) = .31, *p* = .76, and subjective readability, *t*(55) = 1.93, *p* = .059 on likeability, were found not to be significant. However, the effect of expressiveness on likeability was found to be significant, *t*(56) = 3.09, *p* = .003, with these four predictors accounting for 67% of the sample variance (R^2^ = .67).The indirect effects of AQ on likeability were as follows: Through expressiveness, (-.31) (.48) = -.146, 95% CIs [-.32, -.03] (significant); through actual readability, (-21) (.05) = -.010, 95% CIs [-.08, .05] (nonsignificant); through subjective readability, (-.07) (.41) = -.03, 95% CIs [-.09, .002] (non-significant); through expressiveness and actual readability, (-31) (.10) (.05) = -.001, 95% CIs [-.02, .01] (nonsignificant); through expressiveness and subjective readability, (-.31) (.62) (.41) = -.08, 95% CIs [-.19, -.001] (significant); through both readabilities, (-.21) (.34) (.41) = -.03, 95% CIs [-.07, -.001] (significant); through expressiveness and both readabilities, (-.31) (.10) (.34) (.41) = -.004, 95% CIs [-.01, .003] (nonsignificant).

## Discussion

The current study aimed to test an association between actual readability and social favourability but then further model this relationship in the context of other explanatory factors including autistic traits, expressiveness, and subjective readability. We hypothesised that the level of autistic traits of the target would impact target social favourability and likeability, potentially directly, but also via the three potential mediating factors of expressiveness, actual readability and subjective readability. The results supported there being a correlation between actual readability and social favourability; however, this appeared to be an indirect relationship that could be explained by other mediating variables in the model. First, we comment on the simple relationships between the variables, then we discuss the findings of the mediation models, before considering the implications and limitations of the research.

In line with Alkhaldi et al. [12], simple correlations revealed associations between readability (subjective and actual), and social favourability and likeability—this relationship maintained for both forms of readability, although it was higher for subjective readability than objectively measured (i.e., ‘actual’) readability. Moreover, the level of autistic traits of the target correlated negatively with all other variables, such that individuals with higher autistic traits were less readable across different scenarios, were rated less expressive and less readable, and were also perceived as less socially favourable and less likeable than individuals with lower autistic traits. These relationships were preserved when the diagnostic status of targets (nine of whom reported having a formal autism diagnosis) was accounted for as a covariate, suggesting that the correlations with autistic traits were not purely driven by the inclusion of a small number of autistic targets within the sample. Instead, they align with findings of [10, 12], which suggest that readability and social favourability are associated transdiagnostically.

Our two mediation models tested potential mediators of the relationship between autistic traits as predictor, and two outcome variables, respectively: social favourability (the average of the four trait scale judgments) and likeability (the number of “like” judgments the target received based on the dichotomous question “Do you like this person?”) As the findings for the two models differed slightly, we will discuss each in turn.

For social favourability, the model was significant but there was no evidence of a direct effect of autistic traits on social favourability; however, the indirect effect of autistic traits via expressiveness was significant, consistent with full mediation of the effect. The impact of autistic traits on social favourability was not significantly mediated by the two readability variables (i.e., actual and perceived), either independently or in combination. However, some further relationships were observed. There was a direct effect of autistic traits on actual readability, and both actual readability and expressiveness impacted subjective readability.

Taken together these findings suggest that autistic traits impact perception of a target in two ways. Firstly, autistic traits impact how expressive the target is (or at least how expressive they appear to be), which, in turn, impacts perceivers judgments about the social favourability of a target. This is in line with previous research indicating that people who are more expressive are rated as both more extraverted and as more attractive [24]. Secondly, autistic traits directly impact how readable a target is. The fact that this relationship was not related to expressiveness is consistent with [15] who reported that autistic targets were less readable but not necessarily less expressive. Sheppard et al. [15] argued that this may indicate that autistic people behave in a way that is no less expressive but that is qualitatively different from non-autistic people, and harder for non-autistic people to interpret. The present study does nevertheless find clear evidence for expressivity being lower in those with higher levels of autistic traits.

There was no direct relationship between readability and social favourability; rather, the relationship (as indicated by correlation) is predominantly explained by covariance with other related factors. Finally, both expressiveness and actual readability independently contribute to subjective judgments of readability—each of these acting as a mediator for AQ score, suggesting a dual pathway from autistic traits to perceived readability. These findings suggest that perceivers do have a sense of whether they were able to correctly read the targets, but that their judgments are also influenced by how expressive the target seemed regardless of whether the perceiver had correctly read them. The findings also contrast with [10] in that subjective readability did not directly relate to social favourability. This may come down to the present study’s explicit inclusion of expressivity in the explanatory model.

Slightly different findings were observed in relation to the outcome variable of likeability. Again, the overall model was significant, and the direct effect of autistic traits on likeability was not significant, consistent with full mediation. However, in this model there were three significant mediation pathways. Firstly, expressiveness was a significant mediator of the relationship between autistic traits and likeability, suggesting that targets with higher autistic traits are less expressive and that this directly impacts perceivers’ judgments of whether or not they like that target. Secondly, autistic traits impacted likeability via expressiveness and subjective readability, and thirdly, via actual readability and subjective readability. Thus, consistent with [10], this model implies that perceivers are more likely to judge that they like a target if they *felt* that target was readable—though note that the direct effect here approached significance (*p* = .059). As in the previous model, both expressiveness and actual readability influenced subjective readability independently with no direct relationship between expressiveness and actual readability. Overall, the fact that there was a significant mediation pathway involving actual readability suggests that readability and likeability are related insofar as perceivers are aware when they have correctly read the target and this impacts their likeability judgments.

The question arises as to why findings differed for the two models given that previous research (and indeed this study) has found strong correlations between social favourability measures and likeability [39]. Social favourability scores were created by combining mean scores on various rating scales of traits whereas likeability scores reflect the answer to a forced-choice question about whether or not the perceiver likes the target. Consequently, likeability is potentially a more direct measure of the perceivers’ own feelings about the target while social favourability judgments could be influenced by how the perceiver thinks people in general would view the target. If this is the case, it might help to explain why subjective impressions of readability impact likeability more strongly than social favourability. Nevertheless, just because certain mediation pathways were significant in one model and not the other, it does not follow that relationships were substantially different–only that they met criteria for significance in one model and not the other.

Before considering the implications of this research we will discuss a few limitations. Perceivers viewed the various samples of the target’s behaviour simultaneously played in a loop on a single screen, to ensure that all measures were based on the same stimuli presented in the same way. While this should lead to more accurate and robust indices of judgments like social favourability, for the readability measure it allowed perceivers to directly compare the same target’s reactions to the four different scenarios, possibly allowing for them to use processes of elimination to identify the correct scenarios. While this could have led to actual readability scores being artificially elevated, we were more concerned with relative levels of readability between targets than absolute levels.

A feature that might seem like a limitation but is actually a strength is that expressiveness, readability, and social favourability judgments were made by different groups of perceivers. This was by design (as in [12]) to eliminate the possibility of carry-over effects between the different ratings tasks caused by demand characteristics. Nevertheless, [10] measured attraction and subjective readability with the same perceivers and reported a direct contingency between trials where the perceiver felt they had read the target correctly and attraction, which could not be assessed in our research. Moreover, as this study was about illuminating the relationship between readability and social favourability of targets, we did not measure every possible variable that could impact social favourability perceptions, such as physical attractiveness of the targets [8, 9].

A further limitation is that we did not measure the level of autistic traits of the perceivers. It has previously been shown that the match in autistic traits between friendship dyads predicts various measures of the quality of relationships [47] so some of the judgments in this study might also be affected by the discrepancy between individual target and perceiver autistic trait levels.

Finally, it is important to emphasise that this study examined individuals with varying levels of autistic traits and we cannot draw any strong conclusions about autistic people from this study (cf. [48]). While the correlations between variables did not substantially change when controlling for autism diagnosis as a covariate, only a minority of the targets (9/60) stated that they had a diagnosis of autism, which is too small to be able to determine whether the same mechanisms are at work among diagnosed autistic individuals.

The findings of this study provide new knowledge that social impressions are influenced by autistic traits across the population, in addition to being influenced by diagnostic (autistic vs non-autistic) status. Previous research shows that autistic people experience increased likelihood of poor mental health, such as depression and anxiety [19], and it has been argued that this might in part be a consequence of being misperceived or perceived negatively by members of wider society [49]. The research reported here suggests that people at the higher end of the autistic traits continuum might be susceptible to similar negative outcomes as those diagnosed with autism. If this is the case, then interventions at the level of society should not merely be based around educating society about autism (and misperceptions thereof) but also at promoting a wider tolerance of diversity of expression regardless of diagnostic status.

In summary, the current research demonstrated an association between how easy a target’s behaviour is to read and how socially favourable/likeable they are judged to be. Mediation models revealed that the target’s level of autistic traits impacts both how readable their behaviour is and how socially favourably they are perceived. The association between autistic traits and social favourability appears to be primarily accounted for by people with lower autistic traits being more expressive than those with higher trait levels, and those who are more expressive are perceived more favourably. However, differences in expressiveness did not account for the association between autistic traits and readability. Instead, it may be that those higher in autistic traits produce behaviour that is less normative so more difficult to interpret. These findings highlight the importance of target autistic traits in various aspects of person perception.

## Supporting information

S1 Table Perceiver reading accuracy for each scenario. (DOCX)
