# Supplementary material for: The relationship between autistic traits, expressiveness, readability and social perceptions
Source: PLoS One. 2024 Mar 28;19(3):e0301003. doi: 10.1371/journal.pone.0301003 (PMC10977699; doi:10.1371/journal.pone.0301003)
Supplement: S1 Table — (DOCX) [file pone.0301003.s001.docx]

**Table S1**

Mean (and standard deviations of) proportion of perceivers answering correctly for each scenario. Chance = 0.25

|  | *M* | *SD* |
| --- | --- | --- |
| Story | 0.48 | 0.14 |
| Negative feedback | 0.42 | 0.15 |
| Gift | 0.64 | 0.20 |
| Debriefing | 0.74 | 0.13 |
